# Glutathione S-transferase omega in the lung and sputum supernatants of COPD patients

**DOI:** 10.1186/1465-9921-8-48

**Published:** 2007-07-06

**Authors:** Terttu H Harju, Mirva J Peltoniemi, Paula H Rytilä, Ylermi Soini, Kaisa M Salmenkivi, Philip G Board, Lloyd W Ruddock, Vuokko L Kinnula

**Affiliations:** 1 Department of Internal Medicine, University of Oulu, Oulu, Finland; 2 Biocenter Oulu and Department of Biochemistry, University of Oulu, Oulu, Finland; 3 Department of Medicine, Division of Allergology, University of Helsinki, Helsinki, Finland; 4 Department of Pathology, Helsinki University Hospital, Helsinki, Finland; 5 John Curtin School of Medical Research, Australian National University, Canberra, Australia; 6 Department of Pathology, Oulu University Hospital, Oulu, Finland; 7 Department of Medicine, Division of Pulmonary Diseases, University of Helsinki and Helsinki University Hospital, Helsinki, Finland; 8 Department of Clinical Pathology and Forensic Medicine, University of Kuopio, Kuopio, Finland

## Abstract

**Background:**

The major contribution to oxidant related lung damage in COPD is from the oxidant/antioxidant imbalance and possibly impaired antioxidant defence. Glutathione (GSH) is one of the most important antioxidants in human lung and lung secretions, but the mechanisms participating in its homeostasis are partly unclear. Glutathione-S-transferase omega (GSTO) is a recently characterized cysteine containing enzyme with the capability to bind and release GSH *in vitro*. GSTO has not been investigated in human lung or lung diseases.

**Methods:**

GSTO1-1 was investigated by immunohistochemistry and Western blot analysis in 72 lung tissue specimens and 40 sputum specimens from non-smokers, smokers and COPD, in bronchoalveolar lavage fluid and in plasma from healthy non-smokers and smokers. It was also examined in human monocytes and bronchial epithelial cells and their culture mediums *in vitro*.

**Results:**

GSTO1-1 was mainly expressed in alveolar macrophages, but it was also found in airway and alveolar epithelium and in extracellular fluids including sputum supernatants, bronchoalveolar lavage fluid, plasma and cell culture mediums. The levels of GSTO1-1 were significantly lower in the sputum supernatants (p = 0.023) and lung homogenates (p = 0.003) of COPD patients than in non-smokers.

**Conclusion:**

GSTO1-1 is abundant in the alveolar macrophages, but it is also present in extracellular fluids and in airway secretions, the levels being decreased in COPD. The clinical significance of GSTO1-1 and its role in regulating GSH homeostasis in airway secretions, however, needs further investigations.

## Background

Several studies suggest the importance of oxidative stress in the pathogenesis of chronic obstructive pulmonary disease (COPD). Cigarette smoke not only contains high levels of oxidants, but it also activates oxidant producing pathways in the lungs [1,2]. The oxidant/antioxidant imbalance present in the lungs of these patients also results from the impaired capacity of the antioxidant/detoxification enzymes to detoxify the harmful reactive oxygen metabolites [3-8]. Very little is known about specific changes in the major antioxidant defence mechanisms in mild or severe COPD.

One of the major antioxidants in human airways is glutathione (GSH) (L-γ-glutamyl-L-cysteinyl-glycine); however the regulatory mechanisms controlling the intra- and extra-cellular concentrations of GSH are not completely understood [9-11]. The rate limiting enzyme in GSH biosynthesis, glutamate cysteine ligase (GCL) is induced by cigarette smoke [12], but controversially shown to either increase or decrease in COPD [5,13,14]. GCL levels alone do not explain the changes observed in the free GSH levels of airways in smokers or COPD [7]. Other enzymes that can participate in GSH homeostasis in the lung and airway secretions include glutathione peroxidases (Gpx); for example Gpx2 is induced in experimental mice model by smoke exposure [15] and Gpx3 is increased in the bronchial epithelium and epithelial lining fluid of smokers [16]. Another additional group of enzymes that is associated with GSH homeostasis in human airways is glutaredoxin (Grx) family of enzymes. The classical member of this family, Grx1, is regulated in bronchial epithelial cells by oxidants and cigarette smoke *in vitro *[8], but has also been shown to be present in the extracellular fluids including sputum supernatants [17-19]. One important function of glutaredoxins is their thioltransferase activity and the subsequent effects on the glutathionylation state of proteins in the lung. It has become apparent that there is another thioltransferase i.e. glutathione-S-transferase omega (GSTO) in mammalian cells which may have potential role in regulating GSH homeostasis. This enzyme belongs to the glutathione-S-transferase family (GST) that detoxify toxic substrates present in tobacco smoke by a GSH-dependent mechanism [20,21]. GSTO contains an N-terminal glutathione-binding domain suggesting its role in the metabolism and maintenance of GSH levels in intact cells [20,22]. Since GSH is one of the major antioxidants of the airways, it can be hypothesized that GSTO may participate in the maintenance of GSH not only intracellularly, but also in the extracellular space and this may be modulated by oxidative stress.

The present study was undertaken 1) to investigate the cell specific distribution and expression of GSTO1-1 in healthy human lung, 2) to compare the GSTO1-1 expression patterns in the lung of non-smokers, smokers without obstruction and smokers with variable severities of COPD, 3) to assess whether GSTO1-1 is associated with COPD severity and 4) to analyze whether GSTO1-1 can be detected in airway secretions/induced sputum supernatants, bronchoalveolar lavage fluid (BALF) or plasma.

## Methods

### Tissue, induced sputum, bronchoalveolar lavage and plasma specimens

Lung tissue specimens from 72 patients including 26 current smokers with COPD, 22 current smokers with normal lung function, 16 life-long non-smokers undergoing resection for lung tumour (local carcinoma or hamartoma) and 8 ex-smokers with severe COPD undergoing lung transplantation were collected for immunohistochemical studies from the archives of the Departments of Pathology, Oulu University Hospital or Helsinki University Hospital. COPD was defined on the basis of preoperative lung function: FEV1/FVC less than 70% and no reversibility (bronchodilatation effect less than 12%) according to GOLD criteria [23]. All lung transplant patients with stage IV COPD were receiving inhaled corticosteroid therapy. All smokers were current smokers with the exception of lung transplant patients, who were all ex-smokers. The clinical characteristics of the patients in the immunohistochemical studies are shown in Table 1. Tissues for the Western analyses had been frozen immediately in liquid nitrogen after the surgery, and homogenized in ice cold phosphate buffered saline (PBS); the clinical characteristics of these patients are presented in Table 2. A total of 8 patients in the COPD group (two patients with stage I-II COPD and 6 lung transplant cases with stage IV COPD) were receiving inhaled corticosteroid therapy. None of the subjects had received N-acetylcysteine treatment.

**Table 1:** The characteristics of the patients in the immunohistochemistry studies

	Non-smoker N = 16	Smoker N = 22	COPD N = 34	p-value
Age, years	65 (13)	63 (8)	62 (9)	0.543
Sex M:F	8:8	17:5	27:7	0.103
Pack-years	0	46 (19)	38 (13)*	0.000
FEV1 %predicted	98 (15)	90 (10)	55 (23)	0.000
FEV1/FVC %	86 (9)	83 (11)	56 (15)	0.000
MEF50 %pred	94 (24)	80 (37)	34 (21)	0.000
DCO %pred	91 (15)	78 (14)	64 (27)	0.004
DCO/VA %pred	89 (11)	83 (12)	72 (24)	0.035

Mean (SD)

*In post-hoc comparison only the alpha-1-antitrypsin group differed from smokers and other COPD-groups with mean pack-years of 18 (SD 9) years.

**Table 2:** The characteristics of the patients in Western blotting for whole lung homogenates

	Non-smoker N = 9	Smoker N = 5	COPD N = 17	p-value
Age, years	62 (13)	60 (3)	61 (9)	0.0917
Sex M:F	4:5	4:1	13:4	0.350
Pack-years	0	41 (14)	35 (15)*	0.000
FEV1 %predicted	99 (20)	84 (14)	50 (28)	0.000
FEV1/FVC %	88 (6)	81 (7)	55 (17)	0.008
MEF50 %pred	94 (29)	84 (61)	37 (20)	0.009
DCO %pred	92 (18)	81 (11)	54 (22)	0.002
DCO/VA %pred	98 (18)	88 (1)	61 (22)	0.002

Mean (SD)

*In post-hoc comparison no significant differences were found between smokers and the COPD-groups considering pack-years.

Sputum was induced by inhalation of 4.5% hypertonic saline given at 5-minute intervals for a maximum of 20 minutes according to the guidelines of the European Respiratory Society's Task Force [24]. The characteristics of the patients selected for the studies on induced sputum specimens are shown in Table 3.

**Table 3:** The characteristics of the patients in the sputum study

	Non-smoker N = 6	Smoker N = 5	COPD N = 15	p-value
Age, years	59 (3)	52 (6)	63 (8)	0.06
Sex M:F	4:2	4:1	8:7	0.103
Pack-years	0	28 (11)	47 (12)	0.000
FEV1 %predicted	107 (6)	102 (15)	62 (18)	0.001
FEV1/FVC %	83 (5)	82 (5)	64 (10)	0.001
MEF50 %pred	99 (12)	103(12)	31 (19)	0.001
DCO %pred	96(10)	84 (13)	65 (12)	0.001
DCO/VA %pred	101 (12)	102 (6)	76 (17)	0.003

Mean (SD)

Bronchoalveolar lavage (BAL) had been obtained from 3 non-smokers and 3 smokers who had been investigated for minor respiratory symptoms of unknown etiology. Fiberoptic bronchoscopy for sampling BAL fluid was performed under local anaesthesia with lignocaine and the fluid was collected after installation of 10 aliquots of 20 ml from the right middle lobe. The cytocentrifuge preparation indicated a normal cell differential count with over 90% of the cells being macrophages. After centrifugation (400 × g for 15 minutes), the cells and supernatant were collected, frozen and stored at -80 C.

Plasma samples were collected from 4 non-smokers, 4 healthy smokers and 4 patients with stage I-II COPD.

### Cell cultures

Human histiocytic lymphoma (U937) cells were obtained from the American Type Culture Collection [25]. The cells were cultured in RPMI 1640 supplemented with 10% fetal bovine serum, 100 units/ml penicillin, and 100 μg/ml streptomycin. Monocyte-macrophage differentiation was induced by phorbol 12-myristate 13-acetate (PMA) at concentrations of 100 ng/ml. Human non-malignant bronchial epithelial (BEAS-2B) cells (American Type Culture Collection, Rockville, MD, USA) were cultured in Bronchial Epithelial Growth Medium (BEGM) (Clonetics Corporation, Walkersville, MD, USA) and subcultured before reaching confluence.

### Immunohistochemistry and cytochemistry of GSTO1-1 in the tissues and sputum specimens

One tissue block from each patient was selected from peripheral lung. Four-μm sections were cut for immunohistochemical analysis. The sections were deparaffinized in xylene and rehydrated in a descending ethanol series. Endogenous peroxidase was blocked by incubating the sections in 3% hydrogen peroxide in absolute methanol for 15 minutes. The sections were incubated with the primary antibody for GSTO1-1 using a dilution of 1:200. The immunostaining was done using the Histostain-Plus Kit (Zymed Laboratories Inc., San Francisco, CA), and the chromogen was aminoethyl carbazole (AEC) (Zymed Laboratories Inc.). In negative controls, the primary antibody was substituted with phosphate-buffered saline (PBS) or rabbit primary antibody isotype control from Zymed Laboratories Inc.

The number of macrophages was calculated using the Zeiss AxioHOME Morphometry program (Zeiss, Jena, Germany). GSTO-positive macrophages were counted by two techniques and by three investigators, first by calculating the number in 10 high power fields of the specimen (YS) and secondly by using the Zeiss AxioHOME Morphometry program (Zeiss) (PR, KS). Immunoreactivity was also assessed semiquantitatively by grading the staining intensity of the macrophages, bronchial, bronchiolar or alveolar epithelium or vascular endothelium as negative (0), weak (1) or moderate/intense (2) (YS). GSTO1-1 positive and negative cells in the sputum specimens were counted (400 cells/cytospin).

The cytospin samples were treated with Ortho Permeafix (Ortho Diagnostic Systems Inc., UK) and for immunostaining, Zymed ABC Histostain-Plus Kit was used according to the manufacturer's protocol. The samples were incubated with an antibody against GSTO1-1 and negative control samples with Zymed Rabbit Isotype Control and PBS, and stained with AEC (Zymed Laboratories Inc.) and thereafter with Mayer's haematoxylin.

### Western blot analysis

Western blot analysis from tissue homogenates and sputum supernatants was conducted as described earlier [22] with 1:2000–1:5000 dilution of GSTO1-1 antibody. In previous studies from our laboratory and others [17,26,27] β-actin has shown high individual variability, especially in tissue samples from the diseased lung. Instead of using β-actin as a loading control, the protein concentration was measured carefully as triplicates and equal loading was ensured by staining the blotted membranes with Ponceau S (Sigma Aldrich, St. Louis, MO, USA).

### Statistical methods

The statistical analyses were performed with the SPSS for Windows software (SPSS, Chicago, IL, USA). Continuous data were compared using analysis of variance (ANOVA). When ANOVA results indicated that groups differed, post hoc comparisons were performed using two-tailed t-tests. Categorical data were compared using Fisher's exact test designed for small sample groups. P-values less than 0.05 were considered statistically significant. Correlations to lung functions were analyzed with the Pearson correlation test.

### Ethical considerations

The study protocol was approved by the ethical committees of Oulu University Hospital and Helsinki University Hospital and it is in accordance with the ethical standards of the Helsinki declaration of 1975.

## Results

### Immunohistochemistry from the tissue specimens

**Figure 1:**
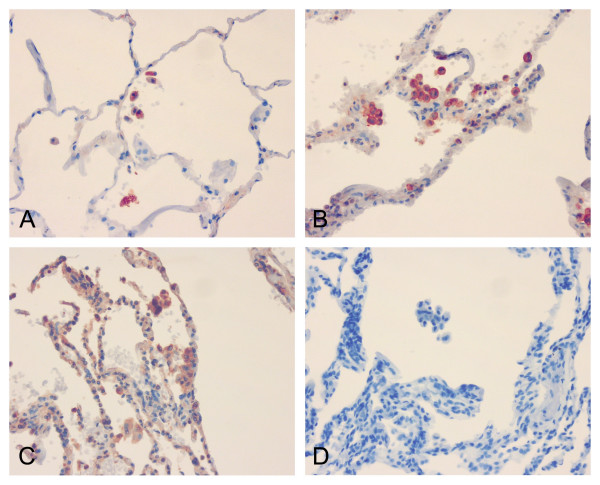
Immunohistochemical staining for GSTO1-1 (1:200) in the peripheral lung of non-smoker (A), COPD stage I-II (B) and COPD stage IV (C). Negative control, COPD stage IV (D). GSTO1-1 was mainly expressed in alveolar macrophages.

**Figure 2:**
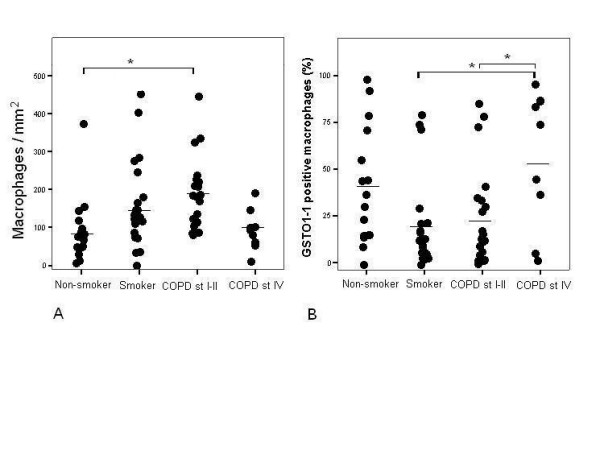
A. The numbers of alveolar macrophages were increased in smokers and in stage I-II COPD compared to non-smokers and stage IV COPD (p = 0.009). The post-hoc comparison was significant at 0.05 level between non-smokers and stage I-II COPD (*). B. The mean percentage of GSTO1-1 positive macrophages was higher in severe stage IV COPD compared to smokers or COPD stage I-II (p = 0.004). The difference between non-smokers and other groups was not statistically significant. The post-hoc comparison was significant at 0.05 level between stage IV COPD and smokers and between stage IV and stage I-II COPD (*).

GSTO1-1 was mainly expressed in alveolar macrophages (Figure 1). One typical feature of COPD is the accumulation of macrophages to the lung. Probably due to the low numbers of the cases and high variability in the numbers of alveolar macrophages, the post-hoc comparison was significant only between non-smokers and stage I-II COPD. (Figure 2A)

**Figure 3:**
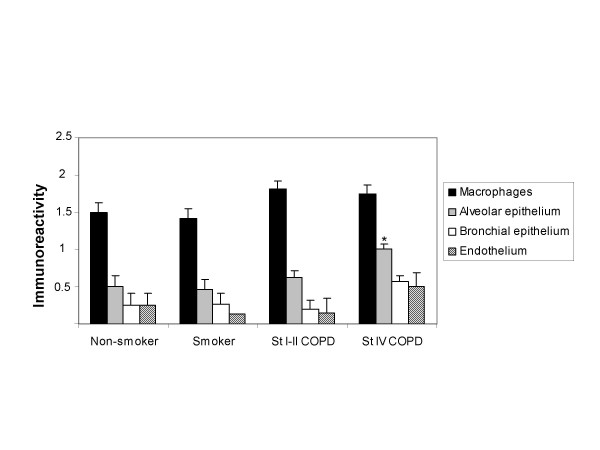
The GSTO1-1 immunoreactivity was most prominent in alveolar macrophages varying from negative to moderate/intense and GSTO1-1 expression was either absent or weak in other cell types. Alveolar epithelium was always positive in stage IV COPD. Bars represent means, error bars standard error of mean.

The number of GSTO1-1 positive macrophages/surface area (mm^2^) were then evaluated, but the percentages of GSTO1-1 positive macrophages did not differ between non-smokers, smokers and COPD-patients (p = 0.085). When the COPD group was divided into stages I-II and IV COPD, the difference between the groups was significant (p = 0.004). The mean (SD) percentage of GSTO1-1 positive macrophages in non-smokers was 40 (30), smokers 19 (25), COPD stage I-II 24 (26) and COPD stage IV 58 (36) and the mean difference was significant at 0.05 level between stage IV COPD and smokers and between stage IV and stage I-II COPD (Figure 2B). The intensity of the GSTO1-1 immunoreactivity in alveolar macrophages varied, being moderate/intense in 8/16 non-smokers, 9/22 smokers and 23/31 COPD-patients. Bronchial and bronchiolar epithelium was either negative or weak (65/69), but by immunohistochemistry the alveolar epithelium was always positive in stage IV COPD. The intensities of GSTO1-1 in various lung cells in healthy and diseased lung including all stages of COPD are shown in Figure 3.

There was a negative correlation between FEV1 and % of GSTO1-1 positive macrophages in all COPD cases (r = -0.533, p = 0.002) and in severe COPD (r = -0.794, p = 0.011). There was no correlation between the pack-years or the dosage of inhaled corticosteroid and percentage of GSTO1-1 positive macrophages.

### Induced sputum extracellular fluids and tissue homogenates

**Figure 4:**
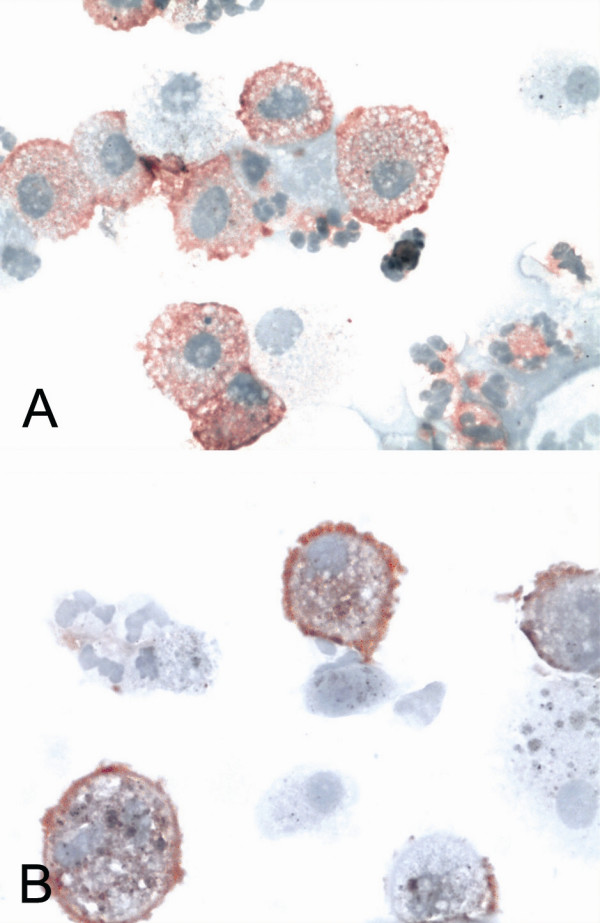
Macrophages in induced sputum exhibited positive GSTO1-1 reactivity. Representative sputum cytospins from a smoker (A) and stage II COPD (B) are shown.

**Figure 5:**
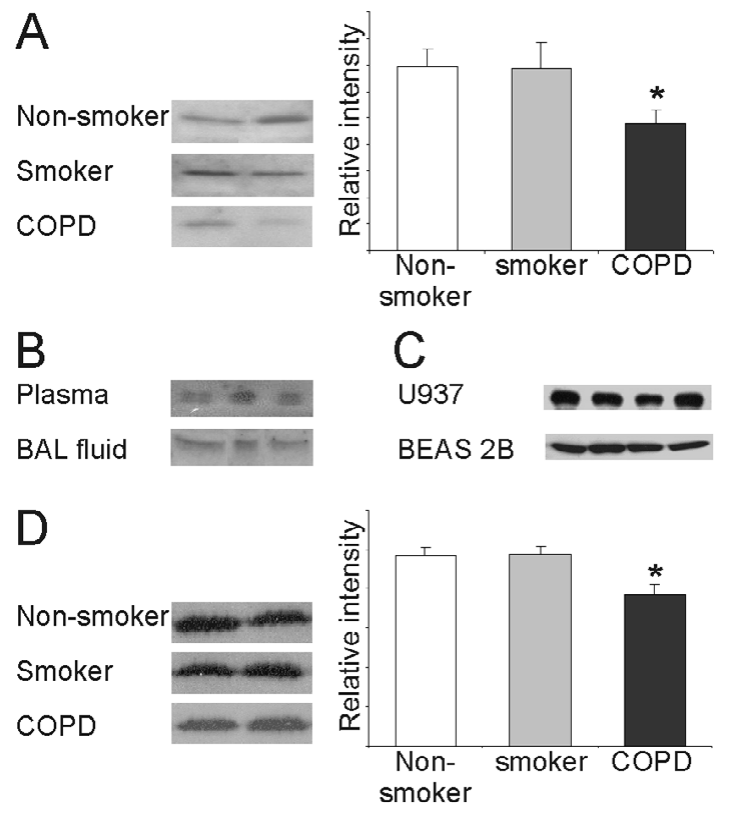
A. Western blotting for GSTO1-1 in induced sputum of non-smokers, smokers and COPD-patients showed an decreased immunoreacitivity in patients with COPD compared to non-smokers (p = 0.023). B. GSTO1-1 could be detected in the plasma samples and bronchoalveolar lavage fluid. Representative Western blots from three non-smokers are shown. C. GSTO1-1 was also expressed in the culture medium of U937 monocytes and BEAS-2B cells. D. Lung tissue homogenates showed higher level of GSTO1-1 in the specimens obtained from non-smokers or smokers with normal lung function when compared to COPD, p = 0.003.

Macrophages in the induced sputum exhibited positive GSTO1-1 reactivity (Figure 4) while neutrophils and lymphocytes were negative. Importantly GSTO1-1 could also be detected in the sputum supernatants while intracellular markers such as β-actin and some other antioxidant enzymes such as manganese superoxide dismutase in these specimens were negative (not shown). Western blot analysis for GSTO1-1 showed decreased levels of GSTO1-1 in the supernatants of COPD patients compared to non-smokers (p = 0.023) (Figure 5A). These results suggest that GSTO1-1 is excreted to the extracellular fluids both in healthy lung and COPD.

To confirm the presence of GSTO1-1 in extracellular fluids, GSTO1-1 was analyzed also from plasma and BALF samples of non-smokers and smokers and from the mediums of the cell cultures (U937 and BEAS2B). GSTO1-1 could be detected in all of samples (representative samples are shown in Figure 5B,5C). Tissue homogenates that contain both lung cells and extracellular matrix, exhibited a lower level of GSTO1-1 in the specimens obtained from the COPD cases compared to the non-smokers and smokers with normal lung function (p = 0.003) (Fig 5D). In Western analysis, there was no correlation between the lung function parameters and relative intensity in the lung homogenates

## Discussion

Glutathione related mechanisms that function both intra- and extracellularly are known to be crucial in the pulmonary defense against oxidants and probably also against cigarette smoke. Here we show that GSTO1-1 has a highly specific localization in the lung, being expressed mainly in alveolar macrophages, but also weakly in other cell types such as airway/alveolar epithelial cells. Importantly GSTO1-1 could also be detected in extracellular fluids including sputum supernatants, BALF, plasma and the cell culture mediums of cultured monocytes and bronchial epithelial cells. This finding strongly supports the idea that the regulation of the GSH homeostasis is not only regulated by intracellular antioxidant enzymes, but is associated with extracellular thiol-modulating proteins that participate in GSH binding and release.

Previously the distribution of GSTO1-1 has been investigated in one human study [21] which showed it to be abundant in a wide range of normal tissues, particularly in the liver but also in the lung (three specimens); in the lung GSTO1-1 could be found only in macrophages. The results of the present study are in line with these findings, but also found GSTO1-1 immunoreactivity in the bronchial and alveolar epithelium. The immunoreactivity was also confirmed in BEAS-2B bronchial epithelial cells in culture.

**Figure 6:**
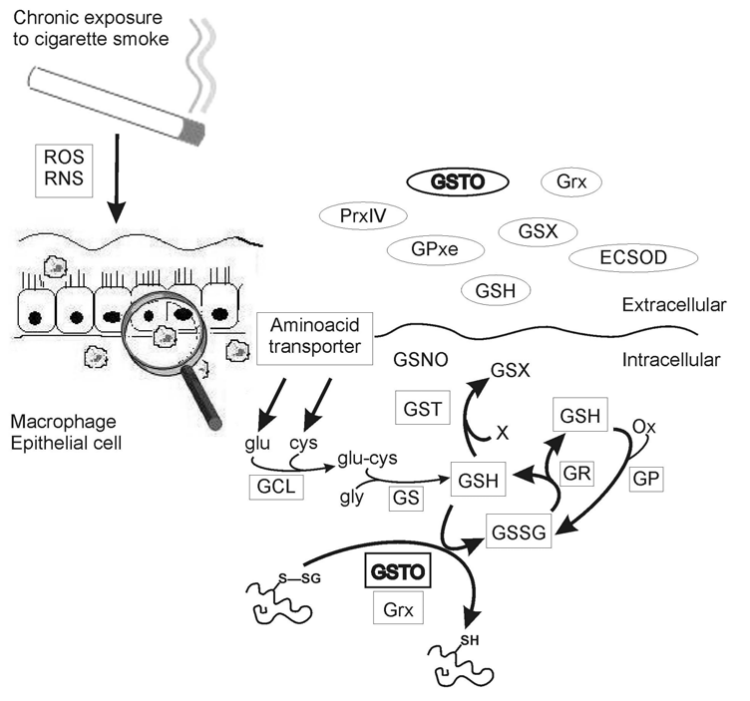
Suggested role of GSTO (glutathione transferase omega) in cigarette smoke induced oxidative stress. Enzymes maintaining GSH homeostasis are important in protecting lung against cigarette smoke induced oxidative stress. GSTO belongs to the glutathione-S-transferase family (GST) that detoxifies toxic substrates present in tobacco smoke by a GSH-dependent mechanism. GSTO contains an N-terminal glutathione-binding domain and is able to bind and release GSH. GSH is one of the major antioxidants in airway secretions, and it can be hypothesized that GSTO participates in the maintenance of GSH homeostasis not only intracellularly but also in the extracellular space. ROS, reactive oxygen species; RNS, reactive nitrogen species; GSH, reduced glutathione; GSSG, oxidized glutathione; GR glutathione reductase; GCL, glutamate cysteine ligase; Grx, glutaredoxin; Prxe, peroxiredoxin, GPXe, glutathione peroxidase (extracellular); GSX, glutathione; ECSOD, extracellular superoxide dismutase; GSNO, nitrosoglutathione; GS, glutathione syntethase.

GSTO1-1 was also detectable in extracellular fluids such as induced sputum supernatants, BALF, plasma and cell culture mediums. Previous studies have already indicated that GSTO1-1 is highly expressed in the liver, suggesting that plasma GSTO1-1 positivity may also be associated with hepatic secretion. All sputum and BALF supernatants were positive for GSTO1-1, but negative for several other markers of intracellular proteins including β-actin. Over 80% of the cells were viable which also argues against any leakage of GSTO1-1 through damaged cell membranes. It is therefore possible that GSTO1-1 is similar to Grx1, in being able to be excreted into the airways where it can participate in the maintenance of GSH homeostasis. GSH levels are increased in the BAL fluid representing epithelial lining fluid (ELF) of cigarette smokers [28], but decreased in the ELF during COPD exacerbations [29]. At present the regulation of GSH in the airway secretions/ELF is far from clear, but GSH synthesis in the ELF is very unlikely to occur. Significant amounts of GSH may be present, however, as protein-GSH mixed disulfides that are known to accumulate during oxidative stress both intracellularly and to the extracellular space [30]. GSTO1-1 is one potential enzyme capable of participating in these reactions: it contains cysteine in its active site where Cys32 can form a disulphide bond with GSH and thereby function as a potential reservoir of GSH during oxidative stress. Previous structural studies have indicated that the active site of GSTO1-1 is relatively open and could potentially accommodate glutathionylated protein structures [31] though the capacity of these reactions is probably diminished in COPD. Overall the regulation of GSH maintenance, binding of GSH to proteins, its oxidation and release is complicated. This study significantly extends earlier observations on these pathways and importance of extracellular fluids in these reactions; summary of these reactions has been gathered to Figure 6.

Tissue studies showed elevated percentage of GSTO1-1 positive macrophages in peripheral lung in severe COPD compared to smokers or stage I-II COPD with negative correlation to lung function parameters. Furthermore, alveolar epithelium was always found to be GSTO1-1 positive in severe COPD. These results may refer to continuing efforts to protect inflamed, remodelled alveolar epithelium and possibly also macrophages against oxidative stress. Overall the synthesis/level of GSTO1-1 may be enhanced in smoker's lung/COPD but together with other antioxidant enzymes is not sufficient to maintain adequate levels of free GSH against oxidative stress.

Tissue homogenates that contain both the cells and matrix showed decreased levels of GSTO1-1 in COPD. These changes in the GSTO1-1 levels in COPD may be partly associated both with decreased levels of macrophages in severe COPD, but also with the presence of all tissue components including the matrix in the lung homogenates and lowered GSTO1-1 levels in the extracellular space.

In summary, this study significantly extends earlier understanding about the antioxidant defence in human lung and extracellular fluids. The important new finding of the presence of GSTO1-1 in extracellular fluids will require further investigation to elucidate the role of GSTO1-1 there since the regulation of GSH levels in these fluids is still poorly understood. The regulation of GSTO is also unknown and there is no literature concerning the effect of oxidative stress on GSTO expression. This is also a critical area requiring research in future investigations.

## Competing interests

The authors declare that they have no competing interests. The study has not been supported by tobacco industry.

## Authors' contributions

THH participated in the design of the study and selection of patient material, performed part of the statistical analysis and drafted the manuscript. MJP carried out the Western blotting studies, participated in analyzing the immunohistochemical data, performed part of the statistical analysis and helped to draft the manuscript. PHR participated in selection and collection of patient material, analyzing the immunohistochemical results and performed part of the statistical analysis. YS and KMS participated in selection of patient material and analyzing the immunohistochemical results. PGB participated in the design of the study, provided the antibody against GSTO1-1 and helped to draft the manuscript. LWR participated in study coordination and helped to draft the manuscript. VLK conceived the study, and participated in its design and coordination and helped to draft the manuscript. All authors have read and approved the final manuscript.
